# Incidents of Practice

**Published:** 1903-05

**Authors:** 


### Incidents of Practice.

Treatment of a Case of "Dry Socket."—A patient for whom a third molar had been extracted by a specialist returned two days afterwards for consultation, complaining of extreme pain in the face, and of the teeth in both maxilla simulating irritation of a pulp. Careful examination excluded the teeth. When the alveolus from which the tooth had been removed was examined, it was found devoid of the normal coagulum, with the bone bare.

A stiff dough-like paste, similar to a suppository, was made of orthoform, combined with oil of sesami and glycerin, with which the socket was filled. This means gave protection to the exposed tissue, as well as nearly immediate relief by the analgesic properties of the orthoform.—L. J.

---

Sensitive Cervical Cavities.—Occasionally patients come to us with one or more cavities at the cervical margin of the teeth. The mouth is unkept, the saliva ropy, the gums puffy with acute gingivitis. There is more or less green stain and accumulation of filth upon the teeth. This condition is very common among dispensary patients. The cavities are very sensitive to prepare for fillings. For many years I have applied iodine to the gums and teeth with good results. In such cases the gums, teeth, and cavities are saturated with iodine every day for from one to two weeks. The patient is required to use the "gum massage brush" to reduce the interstitial gingivitis. Alkaline powder like "vegetol" should be rubbed into the gums and cavities three times a day. If at the end of this period there is slight sensitiveness, local applications may be used at the time of cavity preparation after the rubber dam is applied.—E. S. Talbot, M.D., D.D.S.

